# Generation of a human vascularized 3D airway model replicating native mucosal heterogeneity

**DOI:** 10.3389/fbioe.2026.1761561

**Published:** 2026-03-09

**Authors:** Hannah Kubiza, Julian Gonzalez-Rubio, Stefan Jockenhoevel, Anja Lena Thiebes

**Affiliations:** Department of Biohybrid & Medical Textiles, Institute of Applied Medical Engineering, Helmholtz Institute, RWTH Aachen University, Aachen, Germany

**Keywords:** 3D *in vitro* model, air-liquid interface, cilia, differentiation, human airway epithelium, hydrogel, tri-culture, vascularization

## Abstract

*In vitro* models of the human airway are essential to study respiratory diseases and test potential therapeutics while reducing animal testing. Current models often use two-dimensional culture conditions rather than replicating the physiological 3D environment and do not allow direct cell-cell interactions between the diverse cell types found in the mucosa. Here, we provide a detailed step-by-step instruction for reproducibly generating a complex tri-culture model, which can be used to investigate the human airway environment in health and disease. The model is fabricated by preparing an epithelialized fibrin hydrogel with embedded endothelial and stromal cells. To assure complete differentiation into a mucociliary phenotype, samples are maintained at air-liquid interface (ALI) for 28 days. Afterwards, morphology and functionality can be validated using downstream analysis techniques such as immunohisto- and cytochemistry, electron microscopy, ciliary beating frequency analysis, measurement of mucociliary clearance and RNA isolation. After 4 weeks of maturation, a well-differentiated pseudostratified epithelium comprising basal, multiciliated and secretory cells is developed. We also observe a physiological ciliary beating frequency, mucus production and a functional particle clearance. Inside the hydrogel, endothelial cells form a three-dimensional network of vascular structures. These features make our model ideal for replicating human mucosal heterogeneity, especially compared to airway models using tumor-derived or immortalized cell lines, monocultures or rigid substrates. Hence, this protocol paves the way for fellow researchers to achieve robust airway *in vitro* modeling that can be performed in a standard cell culture lab without the need for extraordinary equipment or specialized expertise.

## 1 Introduction

The airway epithelium represents the first line of defense within the respiratory system. This highly specialized pseudostratified cell layer comprises at least 10 different cell types, the most abundant being basal, multiciliated and secretory cells. (Deprez et al., 2020; Travaglini et al., 2020). The epithelium is supported by its lamina propria, an innervated and vascularized layer of loose connective tissue. Together, both layers form the respiratory mucosa. While the stromal compartment provides nutrients and structural integrity, the epithelial cells ensure airway clearance by removing mucus-entrapped pathogens and particles through coordinated ciliary beating. Both stromal and epithelial airway cells interact with each other and are indispensable for airway homeostasis and regeneration (Zepp and Morrisey, 2019).

Highly prevalent diseases such as asthma, chronic obstructive pulmonary disease (COPD) and lung cancer originate from and act within the airways (Seguin et al., 2022; Wang et al., 2020; Hadzic et al., 2020; Frey et al., 2020). While COPD accounted for 3.3 million deaths, asthma has the highest prevalence with more than 260 million cases in 2019. Also, the incidence of lung cancer and COPD is expected to increase drastically until 2050 (Momtazmanesh et al., 2023; Boers et al., 2023; Sharma, 2022), expanding the socioeconomic burden of airway diseases. Traditionally, airway disease models are almost exclusively using animal-based approaches, despite the increasing evidence that their results are limited in predicting clinical efficacy and safety (Pound and Ritskes-Hoitinga, 2018; Semaniakou et al., 2018; Movia and Prina-Mello, 2020; Movia et al., 2020). Human-derived airway *in vitro* models on the other hand, have emerged as a promising alternative to improve the accuracy of pharmaceutical or infection studies (Rijsbergen et al., 2021; Selo et al., 2021; Cidem et al., 2020). However, the use of simple (monolayer) epithelial cell culture models has been criticized, since they do not account for the physiological complexity of cell interactions within the mucosa. Thus, there is a need for well-differentiated and three-dimensionally engineered *in vitro* tissues and their popularity is rising (Shrestha et al., 2020; Zhou et al., 2023). Motivated by the idea of developing an authentic and responsive *in vitro* model of the airway mucosa, our lab combined years of expertise in the tissue engineering of the respiratory epithelium and vascular structures.

**FIGURE 1:**
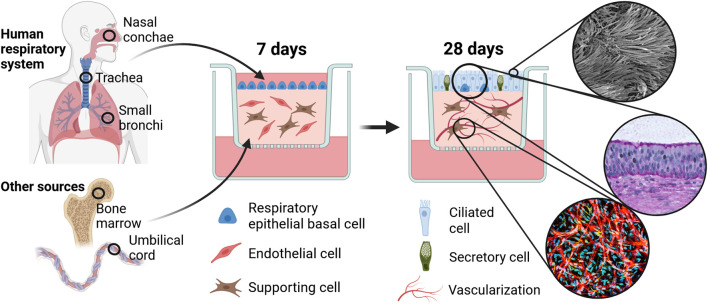
Fabrication of 3D airway *in vitro* model and anticipated results. Primary human respiratory epithelial cells are cultured on top of a fibrin hydrogel matrix enriched with primary supporting and endothelial cells. Tri-cultures are maintained for 28 weeks at air-liquid interface and subsequently analyzed with regard to epithelial maturation into a mucociliary phenotype and the formation of vascular-like structures. Created with BioRender.com.

The result is a complex multi-cell-type *in vitro* airway model based on a hydrogel scaffold (Kreimendahl et al., 2019), which includes three human primary cell types, airway epithelial cells, endothelial cells and supporting stromal cells. The mature tissue comprises a well-differentiated pseudostratified epithelium with a mucociliary phenotype and sprouting vascular-like structures in the fibrin hydrogel below (Figure 1).

The tri-culture is highly versatile and can be used in a wide range of applications. This includes the establishment of disease modeling and drug screening platforms. Lately, our group demonstrated SARS-CoV-2 replication in the model and observed increased ciliation in response to inactivated viral particles (Gonzalez-Rubio et al., 2023). This approach can be expanded to study other common airway diseases, such as asthma and COPD. Current *in vitro* studies mostly employ simplified models, which could be easily adapted to the herein-described system (Stewart et al., 2012; Osei and Hackett, 2020). The model allows the incorporation of additional cell types, like immune cells or airway smooth muscle cells. Moreover, healthy cells can be substituted with those from diseased donors or with genetically engineered iPSC-derived cells to investigate congenital conditions like cystic fibrosis or primary ciliary dyskinesia (Vilà-González et al., 2024; Demchenko et al., 2024). In addition, the 3D hydrogel system enables the incorporation of chemically and mechanically tunable substrates, which can be utilized to reflect tissue remodeling during lung disease progression (Hinz, 2012). Apart from pre-clinical *in vitro* applications, the tri-culture is a very useful tool for the investigation of basic research questions. Being a highly controlled system, it can be easily adapted to isolate specific parameters. The tri-culture might also be of interest within the field of classical tissue engineering for organ replacement. The vision to generate *in vitro* constructs for implantation into patients has been present for more than 30 years (Langer and Vacanti, 1993). However, for complex implants such as the trachea, current options are limited. Building a full-scale airway implant based on the herein-described tissue-engineered mucosa model could provide a solution for patients in need of tracheal or bronchial transplants in the future.

This work describes the complete fabrication of the tri-culture, starting with the preparation of reagents and equipment. We give insights into the culture and expansion of the three required cell types (Steps 1–21). The method section includes detailed instructions on the production of the fibrin hydrogel containing supporting stromal and endothelial cells, as well as respiratory epithelial cells on the apical side (Steps 22–49). We also share our workflow for the maintenance of the tri-culture, which requires a 5-week maturation period (Steps 50–52). Finally, we propose 6 different downstream procedures to characterize and validate the experimental outcomes (Steps 53–134).

## 2 Materials and equipment

### 2.1 Reagents

- 2-Propanol (VWR, Cat. num. 20842.330)
- Acetic acid (Sigma-Aldrich, Cat. num. 33209)
- Agarose (Sigma-Aldrich, Cat. num. A9414)
- Airway Epithelial Cell Growth Medium (AECGM; PromoCell, Cat. num. C-21060)
- Amphotericin B, 250 μg/mL (PAN-Biotech, Cat. num. P06-01005S)
- Bovine serum albumin (Sigma-Aldrich, Cat. num. A9418)
- Calcium chloride (CaCl_2_; Sigma-Aldrich, Cat. num. C-3881)
- Chloroform (VWR, Cat. num. C22711.260)
- Ciprofloxacin Kabi 100 mg/50 mL (Fresenius Kabi)
- DAPI (Carl Roth, Cat. num. 6,335.1)
- Dulbecco’s Modified Eagle Medium, low glucose, pyruvate (DMEM; Gibco, Cat. num. 11885084)
- Dulbecco’s Phosphate-buffered Saline, no calcium, no magnesium (DPBS; Gibco, Cat. num. 14190250)
- Ethanol absolute (VWR, Cat. num. 20816.367)
- Ethylenediamine tetraacetic acid (EDTA; Roth, Cat. num. 8040.2)
- Endothelial Cell Growth Medium 2 (EGM2; PromoCell, Cat. num. C-39211)
- Fetal Bovine Serum (FBS; Gibco, Cat. num. 10270106)
- Fibrinogen from Human Plasma (Sigma-Aldrich, Cat. num. 341578-500 MG)
- Fluoresbrite YG Microspheres 0.50 µm (Polysciences, Cat. num. 17152)
- Gelatine from Bovine Skin (Sigma-Aldrich, Cat. num. G9391)
- Glutaraldehyde 25%, EM grade (Agar Scientific, Cat. num. AGR1010)
- Isoproterenol hydrochloride (Sigma-Aldrich, Cat. num. I6504)
- Keratinocyte SFM (1X) Kit (KSFM; Gibco, Cat. num. 17005042)
- Mesenpan (PAN-Biotech, Cat. num. P08-50400K)
- MucilAir Medium (MucilAir; Epithelix, Cat. num. EP05MM)
- Penicillin-Streptomycin (10,000 U/mL) (Gibco, Cat. num. 15140122)
- Penicillin-Streptomycin/Amphotericin B Mix (ABM; PAN-Biotech, Cat. num. P06-07300)
- Potassium chloride (KCl; Merck, Cat. num. 1.04936.0500)
- Sodium azide (Roth, Cat. Num. K305.1)
- Sodium chloride (NaCl; Sigma-Aldrich, Cat. num. S9625)
- Sodium hydrogen carbonate (Roth, Cat. num. 6885.1)
- Sorensen’s Phosphate Buffer (EMS, Cat. num. 11600-05)
- Thrombin from Bovine Plasma (Cat. num. T4648-1KU)
- Tranexamic acid (TXA; Carinopharm, Cat. num. PZN 10816984)
- TRI Reagent Solution (Invitrogen, Cat. num. AM9738)
- Tris-base (Trizma Base, Sigma-Aldrich, Cat. num. T6066)
- Tris-hydrochloride (Trizma HCl; Sigma-Aldrich, Cat. num. T3253)
- Trypan blue (Gibco, Cat. num. 15250061)
- Ultrapure water (From Millipore Milli-Q lab water system or similar)

### 2.2 Consumables

- Aluminum foil
- Cell culture flask, 550 mL, 175 cm^2^, PS (Greiner, Cat. num. 660175)
- Cell culture insert, 12 mm Transwell with 0.4 µm Pore Polyester Membrane Insert, Sterile (Corning, Cat. num. 3460)
- Chambered coverslip, µ-Slide 8 Well for microscopy (Ibidi, Cat. num. 80826)
- Deep-well plate, ThinCert Plate, 12 Well, PS, Lid, Sterile (Greiner, Cat. num. 665110)
- Dialysis membrane, 6–8 kD molecular weight cut-off (Repligen, Cat. num. 132665)
- Dialysis tubing closures Spectra/Por, PP (e.g., Repligen)
- Embedding cassettes (Simport, Cat. num. M499-12)
- Embedding molds (Tissue-Tek Cryomold, Sakura Finetek, Cat. num. 25608-916)
- Low-protein binding tubes (Eppendorf, Cat. num. 525-0134)
- Scalpel, pointy (Nr. 11) and round (Nr. 23) tips (Feather, Cat. num. 160080 and 161635)
- Screw cap tubes, 15 mL and 50 mL (Sarstedt, Cat. num. 62.547.205 and 62.554.100)
- Sealing film (Parafilm, Carl Roth, CNP8.1)
- Sponge for embedding cassettes (Carl Roth, Cat. num. TT56.1)
- Sterile filter, 0.2 μm (Corning, Cat. num. 431219)
- Syringe, 20 mL, sterile (Braun, Cat. num. 8728615)

### 2.3 Equipment

- Aluminum cooling tray
- Analytical scale (e.g., Sartorius, CPA 5201)
- Autoclave (e.g., Systec, DE-23)
- Borosilicate bottle 250 mL (e.g., VWR, Borosilicate 3.3)
- Brightfield microscope, inverted (e.g., Nikon, Eclipse Ti)
- Cell counting device (e.g., Neubauer chamber)
- Dehydration device (e.g., Leica, TP1020)
- Fluorescence microscope, inverted (e.g., Nikon, Eclipse Ti)
- Fluorescence microscope, upright (e.g., Zeiss, Axio Zoom. V16)
- Freezer (−20 °C)
- Freezer (−80 °C)
- Fridge (4 °C)
- High-speed camera (e.g., The Imaging Source, DMK 33UP1300)
- Magnetic stirrer hot plate (e.g., Heidolph, MR Hei-Standard)
- Magnetic stirring bars
- Microtome (e.g., pfm, Slide 2003)
- Nitrogen tank for cell storage (e.g., ARPEGE, ARPEGE110N-L-1)
- Paraffin embedding station (e.g., Leica, Histo Core Arcadia H)
- pH meter (e.g., Hanna instruments, 2211)
- Pipette controller (e.g., VWR, PipetBoy)
- Spectral photometer (e.g., ThermoScientific, Nanodrop One)
- Water purification system (e.g., Millipore, Milli-Q Direct Water Purification System)

### 2.4 Formulations

#### 2.4.1 2% sodium bicarbonate, 1 mM EDTA solution

Add 40 g of sodium bicarbonate, 585 g of EDTA and 2 L of ultrapure water to a 5 L glass beaker. Stir using a magnetic stirring plate until ingredients are completely dissolved.

#### 2.4.2 0.1% sodium azide solution

For 500 mL, add 500 mg of sodium azide to 500 mL of ultrapure water. Store at 4 °C for up to 1 year.

#### 2.4.3 Tris-buffered saline (TBS) solution

For 1 L solution, combine 4.36 g Tris HCl, 0.64 g Tris Base, 8 g NaCl and 0.2 g KCl in ultrapure water. Add a stirring bar and mix on a magnetic stirring plate until the salts are completely dissolved. Measure pH and while stirring, adjust pH to 7.5 by slowly adding drop by drop either 1 M NaOH solution if the pH is too low, or 1 M HCl solution if the pH is too high. Sterilize using a 0.2-μm filter.

Critical: Sterile filtering is preferred over autoclaving since every heating cycle results in the loss of water volume, which can alter the salt concentration.

#### 2.4.4 Airway epithelial cell growth medium (AECGM)

For AECGM, combine basal medium with provided supplements (final supplement concentration: 0.004 mL/mL bovine pituitary extract, 10 ng/mL epidermal growth factor, 5 μg/mL insulin, 0.5 μg/mL hydrocortisone, 0.5 μg/mL epinephrine, 6.7 ng/mL triiodo-L-thyronine, 10 μg/mL transferrin, 0.1 ng/mL retinoic acid), add 500 µL of 40 mg/mL gentamicin, add 5 mL Penicillin-Streptomycin/Amphotericin B Mix (1%). Store protected from light at 4 °C for up to 6 weeks.

#### 2.4.5 Keratinocyte-serum-free medium (KSFM)

For preparation of KSFM, combine basal medium with supplements (final supplement concentration: 5 μg/L human recombinant epidermal growth factor, 50 mg/L bovine pituitary extract), 1 µM isoproterenol, 100 U/mL penicillin, 100 μg/mL streptomycin, 2.5 μg/mL amphotericin B and 0.01 mg/mL ciprofloxacin. Store protected from light at 4 °C for up to 6 weeks.

#### 2.4.6 Endothelial cell growth medium 2 (EGM2)

Prepare EGM2 by combining endothelial cell basal medium with the provided supplements (final supplement concentration: 0.02 mL/mL fetal calf serum, 5 ng/mL epidermal growth factor, 10 ng/mL basic fibroblast growth factor, 20 ng/mL insulin-like growth factor, 0.5 ng/mL vascular endothelial growth factor 165, 1 μg/mL ascorbic acid, 22.5 μg/mL heparin, 0.2 μg/mL hydrocortisone), add 500 µL of 40 mg/mL gentamicin, add 5 mL Penicillin-Streptomycin/Amphotericin B Mix (1%). Store in the dark at 4 °C for up to 6 weeks.

#### 2.4.7 Gelatine coating solution

For ∼100 mL coating solution, add 2 g gelatine to a borosilicate bottle with 100 mL ultrapure water. Add a stirring bar and place on a magnetic stirrer with a hot plate at 60 °C. Leave to mix until the solution becomes clear (approx. 15 min). Sterilize solution in an autoclave using a liquids program, not screwing the lid completely to allow water vapor to enter. After the program is finished, allow the gelatine to cool down until the bottle can be touched (40 °C–50 °C) and aliquot 5 mL coating solution into sterile 15 mL screw cap tubes. 5 mL is sufficient to coat two T175 cell culture flasks. Store aliquots at 4 °C tightly closed for up to 2 months.

#### 2.4.8 Complete DMEM

Prepare complete DMEM by adding 50 mL FBS (10%) and 5 mL Penicillin-Streptomycin/Amphotericin B Mix (1%) to 500 mL DMEM. Store at 4 °C.

#### 2.4.9 Mesenpan

Prepare Mesenpan by combining 500 mL Mesenpan basal medium with 5 mL of provided supplements (1%), 10 mL FBS (2%) and 5 mL of Penicillin-Streptomycin/Amphotericin B Mix (1%).

#### 2.4.10 CaCl_2_ solution, 25 mM

For CaCl_2_ solution, add 0.3675 g of CaCl_2_ to 100 mL of TBS. Sterilize the solution using a 0.2-μm filter. Store at room temperature for up to 6 months.

Critical: Calcium chloride powder is highly hygroscopic. Keep the container tightly closed and open only briefly to remove the desired quantity. Measure immediately since the absorbed water will quickly increase weight. In case of hydration of the stock calcium chloride after extended air contact time, moisture can be removed by heating up to 200 °C in a borosilicate beaker for at least 2 hours. However, it is preferred to dispose of it and purchase a new dry batch.

#### 2.4.11 Thrombin solution

Prepare 40 IU/mL thrombin stock solution by thawing lyophilized thrombin at room temperature. Dissolve powder in 10 mL TBS. Carefully mix without using a pipette. Transfer solution to a sterile beaker. Rinse thrombin vial with another 15 mL TBS. Sterile filter using a 0.2-µm filter, aliquot in appropriate volumes in low-protein binding tubes, store at −20 °C, use within 1 year.

#### 2.4.12 Fibrinogen stock solution

Place dry and non-reconstituted dialysis membranes in 5 L glass beaker with 2 L of 2% sodium bicarbonate, 1 mM EDTA solution (see 2.4.1). Transfer the beaker to a 60 °C water bath and incubate for 3–4 h. Rinse inside and outside surface thoroughly with demineralized water, followed by an additional washing step with ultrapure water. Cut membranes into pieces of 50 cm length and remove trapped air by squeezing gently. Place membranes in 0.1% sodium azide solution (see 2.4.2) and store for up to 1 year at 4 °C. Prior to usage, flush membranes twice with demineralized water. Collect sodium azide waste separately and dispose of it appropriately. Thaw lyophilized fibrinogen for 1.5 h at room temperature. Slowly add 39 mL ultrapure water to powder. After 4 h, fibrinogen is dissolved. If clumps remain, carefully rotate vial to solve them. Close one end of the dialysis membrane tube with a tubing closure. Transfer fibrinogen to dialysis membrane. Fix the other end with a tubing closure. Place loaded membrane in 4 L TBS buffer (see 2.4.3) in a beaker and stir slowly at 150 rpm overnight. Pour dialyzed fibrinogen solution in a clean beaker. Dropwise sterile filter with a 0.2-µm filter, change filter occasionally if necessary. Determine fibrinogen concentration by photometric absorption at 280 nm. Aliquot in ready-to-use amounts and store at −80 °C. Fibrinogen can be stored for at least 1 year.

Critical: Do not shake or invert freshly dissolved fibrinogen solution to avoid precipitation.

Tip: Purification of commercially available fibrinogen protein is highly recommended. Dialysis against a membrane with a specific molecular cut-off removes salts and albumin, both known to have anti-coagulative effects (Sharma et al., 2015). This improves the thrombin-initiated polymerization and thereby the mechanical properties of the resulting hydrogel.

#### 2.4.13 Proliferation medium (ProM)

For 3D culture proliferation medium, combine 250 mL AECGM, 250 mL EGM2 and 800 µL tranexamic acid (0.16%). Store light protected at 4 °C for up to 6 weeks.

#### 2.4.14 Differentiation medium (DiffM)

For 3D culture differentiation medium, combine 250 mL MucilAir medium, 250 mL EGM2 and 800 µL tranexamic acid (0.16%). Store up to 6 weeks at 4 °C, protected from light.

Critical: Cover the bottle from the light, since crucial supplements for epithelial differentiation such as retinoic acid are photosensitive. Do not warm the whole medium container. Instead, prepare aliquots to avoid supplement degradation from repeated warming-cooling cycles.

#### 2.4.15 Fluorescent beads suspension

Fluorescent beads (Fluoresbrite YG Microspheres 0.50 µm, Polysciences) were diluted 1:1000 using DiffM.

#### 2.4.16 Carnoy’s fixative

To prepare 1 L fixative solution, add 600 mL ethanol, 300 mL chloroform and 100 mL glacial acetic acid in a beaker. Cover the beaker with aluminum foil, since chloroform is light-sensitive.

Caution: The chemical vapors are highly toxic; perform all steps in a chemical fume hood with proper personal protective equipment. In addition, use only glassware when handling chloroform, since it dissolves most types of plastic upon contact.

Critical: Carnoy’s solution is not stable, prepare freshly.

#### 2.4.17 Agarose solution (2%)

For 2% agarose solution dissolve 4 g agarose powder in 200 mL ultrapure water. Use a magnetic stirrer to remove agarose clots. Boil solution for several minutes in microwave until agarose is completely dissolved. Store at 4 °C. Before every use, heat up and boil in microwave for several minutes until mix is liquified again.

Caution: Hot agarose can become superheated and overboil. Wear protective equipment including safety goggles.

#### 2.4.18 Blocking solution (3%)

For bovine serum albumin (BSA) blocking solution, add 1.5 g BSA to 50 mL PBS. The solution can be stored at 4 °C for up to 2 weeks.

## 3 Methods

### 3.1 Primary human airway epithelial cell culture

Primary human airway epithelial cells were isolated from nasal, tracheal or bronchial tissue. Excess nasal conchae tissue was received from the Clinic for Otorhinolaryngology (RWTH Aachen University Hospital, Germany). Cells were isolated, expanded in AECGM (see 2.4.4) and cryopreserved in passage 0 (Kreimendahl et al., 2019). Tracheal resections from healthy donors of lung transplants were provided by the Department Clinic for Thoracic and Cardiovascular Surgery (University Hospital Essen, Germany). Donors were selected according to the Eurotransplant guidelines. Isolation and expansion of tracheal epithelial cells was performed by the was performed by the Department of Pulmonary Medicine (University Medical Center Essen, Ruhrlandklinik, Germany) as described recently (Barbet et al., 2024). Cells were maintained until passage 0 in KSFM (see 2.4.5), followed by long-term storage in liquid nitrogen. Macroscopically normal bronchial tissue from lung resection surgeries of lung cancer patients was provided by the Institute of Pathology (RWTH Aachen University Hospital). Cells in passage 0 were cultured in AECGM and subsequently stored in liquid nitrogen. The final tri-culture was prepared with passage 1 airway epithelial cells. Epithelial cell donors were routinely screened regarding their mucociliary differentiation potential using the here described method. All experiments were conducted with at least three biological replicates (donors).

Tip: The tri-culture can be successfully prepared using nasal, tracheal or bronchial airway epithelial cells. Depending on the research question, cells from specific regions of the airways might be appropriate. Moreover, primary airway epithelial cells are commercially available.

### 3.2 Primary human endothelial cell culture

Primary human endothelial cells were obtained from human umbilical cord veins provided by the Clinic for Gynecology and Obstetrics (RWTH Aachen University Hospital). The isolation was performed as described previously (Menzel et al., 2017; Kreimendahl et al., 2019). During culture, cells were maintained in EGM2 (see 2.4.6). For long-term storage, cells were stored in liquid nitrogen. In the final tri-culture, only cells in passage 4 or lower were used. All experiments were conducted with at least three biological replicates (donors).

#### 3.2.1 Coating of cell culture flasks with gelatine

Timing 45 min.

1. Warm up 5 mL of gelatine solution (see 2.4.7) for 5–10 min in water bath (37°C).
2. Add 2.5 mL per T175 flask and agitate carefully to distribute the solution evenly.
3. Remove leftovers of gelatine solution with a vacuum pump.
4. Leave flasks to dry under the hood for 30 min with the lid closed.
5. Flasks should preferably be used directly after preparation but can be stored for up to 4 weeks at 4 °C sealed with Parafilm.

#### 3.2.2 Thawing of endothelial cells

Timing 20 min.

1. Thaw a vial of endothelial cells (1 × 10^6^) in a 37 °C water bath for 3 minutes.
2. Transfer content of vial into 25 mL of complete DMEM (see 2.4.8).
3. Centrifuge for 5 min at 500 g.
4. Remove supernatant and resuspend in 20 mL of EGM2.
5. Transfer to gelatine-coated T175 cell culture flask (equivalent to a seeding density of 5.700 cells/cm^2^).
6. Place in a humidified incubator at 37 °C and 5% CO_2_.

#### 3.2.3 Expansion of endothelial cells

Timing 30 min; expansion ∼1 week.

1. Change medium every 3–4 days.
2. Culture until cells reach 70%–80% confluency.

### 3.3 Primary human stromal cell culture

Primary human stromal cells were isolated from tracheal-, bronchial tissue or bone marrow. Tracheal resections from healthy lung transplants were provided by the Department Clinic for Thoracic and Cardiovascular Surgery (University Hospital Essen) (see 3.1). Bronchial rings were provided by the Institute of Pathology (RWTH Aachen University Hospital) (see 3.1). Cells were isolated as reported recently and expanded in complete DMEM (Gonzalez-Rubio et al., 2023). Bone marrow-derived stromal cells were isolated from femoral heads provided by the Clinic for Orthopedic, Trauma and Reconstructive Surgery (RWTH Aachen University Hospital) (Luengen et al., 2022; Kniebs et al., 2020) and maintained in Mesenpan (see 2.4.9). Mesenchymal stromal cell identity was regularly confirmed by flow cytometry for positive (CD90, CD73 and CD105) and negative (CD45, CD34, CD11b, CD79A and HLA-DR) expression of surface molecules (Dominici et al., 2006). Both tracheal and bone marrow-derived stromal cells were cryopreserved in liquid nitrogen. In the final tri-culture, only cells in passage 6 or lower were used. All experiments were performed with at least three biological replicates (donors).

#### 3.3.1 Thawing of stromal cells

Timing 20 min.

1. Thaw a vial of stromal cells (2 × 10^5^ cells) in a 37 °C water bath for 3 minutes.
2. Transfer content of vial into 25 mL of complete DMEM.
3. Centrifuge for 5 min at 500 g.
4. Remove supernatant and resuspend in 20 mL of complete DMEM/Mesenpan.
5. Transfer to T175 cell culture flask (equivalent to a seeding density of 1.200 cells/cm^2^).
6. Place in a humidified incubator at 37 °C and 5% CO_2_.

#### 3.3.2 Expansion of stromal cells

Timing 30 min; expansion ∼1 week.

1. Change medium every 3–4 days.
2. Culture until cells reach 70%–80% confluency.

### 3.4 Preparation of cell-laden hydrogel

After expansion and collection of stromal and endothelial cells, both cell suspensions, TBS, calcium chloride (see 2.4.10), thrombin (see 2.4.11) and fibrinogen (see 2.4.12), were used to prepare component A and component B (see Table 1). Prior to mixing, all ingredients were kept on ice. Both components were combined inside a cell culture insert (12 mm Transwell with 0.4 µm Pore Polyester Membrane Insert, Corning) to produce a soft, viscoelastic gel with a final fibrinogen concentration of 5 mg/mL. Blending was performed in a fast and precise manner (see Supplementary Video 1 for detailed procedure). After 40 min of incubation at 37 °C, respiratory epithelial cells were added to the luminal side of the fibrin gel.

**TABLE 1:** Composition of the fibrin hydrogel components before and after mixing, including amount per gel and initial/final concentration.

Hydrogel component	Ingredients	Quantity (per gel)	Initial concentration	Final concentration
Component A	Fibrinogen	100 µL	10 mg/mL	5 mg/mL
Component B	Thrombin	15 µL	40 IU/mL	3 IU/mL
	CaCl_2_	30 µL	25 mM	3.75 mM
	Stromal cell solution	27.5 µL	21.81 × 10^6^ cells/mL	3 × 10^6^ cells/mL
	Endothelial cell solution	27.5 µL	21.81 × 10^6^ cells/mL	3 × 10^6^ cells/mL

#### 3.4.1 Preparation of hydrogel components

Timing 30 min.

1. Thaw aliquots of fibrinogen and thrombin on ice.
2. Dilute fibrinogen stock solution with TBS to a concentration of 10 mg/mL (= component A).
3. To prepare component B, start by combining thrombin and CaCl_2_. Store on ice until further use. The final concentration of each component can be seen in Table 1.

Critical: Use only TBS instead of other buffers such as PBS. Phosphates will hijack the calcium ions of the solution and inhibit fibrin polymerization.

#### 3.4.2 Preparation of cell suspensions

Timing 1.5 h.

1. Rinse stromal cells with 20 mL of PBS.
2. Add 7 mL of 0.05% trypsin.
3. Incubate for 5 min at 37 °C.
4. Deactivate trypsin with 14 mL of complete DMEM.
5. Determine cell number.
6. Centrifuge at 500 *g* for 5 min.
7. Resuspend cell pellet in appropriate volume of ProM.
8. Determine number of viable cells (e.g., trypan blue staining in Neubauer chamber).
9. Dilute cell suspension to achieve an initial concentration of 21.81 × 10^6^ cells/mL in ProM (see 2.4.13).
10. Repeat steps 25.-33. for endothelial cells.

#### 3.4.3 Hydrogel preparation

Timing 1.5 h.

1. Add 27.5 µL of both cell suspensions to thrombin/CaCl_2_ mixture.
2. Place standard 12-well plate with inserts on aluminum cooling tray.
3. Add 100 μL of component A solution to the required number of inserts.
4. Add 100 μL of component B solution to each of the inserts, quickly mix both solutions by pipetting up and down three times.

Critical: Once fibrinogen and thrombin are combined, gelling takes place within seconds. It is recommended to first practice the procedure using non-sterile, cell-free material.

Critical: Careful pipetting is needed to avoid formation of bubbles. To assure thorough distribution of the gel inside the insert, first spread component A using a pipette tip over the whole membrane surface. Afterwards add component B.

1. Incubate the gels for 40 min at 37 °C until they fully polymerize.

Critical: Fibrin hydrogels are sensitive before fully polymerized. Plates with gels must be moved slowly and placed only on leveled surfaces.

1. Transfer inserts to deep well plate.
2. Add 4 mL of ProM in the well to basal side of membrane.

Tip: Researchers are free to explore different hydrogel compositions to fine-tune their protocol. Also, insert membrane properties like pore size, pore density, material and optical properties should be taken into account. The pore size should not exceed nucleus size to prevent migration into the basal compartment. Pore density should be high enough to ensure a sufficient supply of nutrients to the hydrogel. However, a too high pore density might hinder both brightfield and fluorescent microscopy.

#### 3.4.4 Seeding of respiratory epithelial cells

Timing 30 min.

1. Thaw a vial of P0 primary airway epithelial cells in a water bath at 37 °C for two to 3 minutes
2. Transfer epithelial cell suspension into 25 mL of complete DMEM.
3. Centrifuge at 200 *g* for 5 min.
4. Resuspend pellet in appropriate amount of ProM.

Critical: Airway epithelial cells are very sensitive and must be handled with special care. To reduce cell death and failing differentiation, decrease the speed of the pipette controller to the minimum, avoid creating bubbles as well as forcefully propelling the cell suspension against the vessel walls.

1. Determine number of viable cells (e.g., trypan blue staining in Neubauer chamber).
2. Dilute cell suspension to a concentration of 1.8 × 10^5^ cells/mL in ProM.
3. Transfer 0.5 mL cell suspension on top of the hydrogel to achieve a concentration of approximately 8 × 10^4^ cells/cm^2^.
4. Fill empty wells of plate with sterile water to reduce medium evaporation.

Tip: The fibrin gel with stromal and endothelial cells can be maintained in ProM overnight before adding the epithelial cells. This might be beneficial when preparing many samples.

Tip: Preferably, store cultures in infrequently used incubators to minimize cross-contamination and the fluctuations in CO_2_ level and temperature that occur upon opening.

### 3.5 Maturation of tri-culture

In the first week of culture, samples were maintained in a submerged configuration to achieve epithelial confluency. Afterwards, differentiation was initiated by switching to differentiation medium and by allowing direct contact of the epithelial cells with humidified air. Samples were allowed to mature for 28 days. The frequency of medium changes was adjusted to provide a regular supply of fresh nutrients, while avoiding depletion of self-produced growth factors. Deep-well plates (12-well ThinCert Plate, Greiner) with a larger reservoir for basal medium were used to further reduce the frequency of medium changes. At all times, the medium was supplemented with the antifibrinolytic tranexamic acid (Carinopharm) to prevent fibrin gel digestion within the course of a few days (Cholewinski et al., 2009), and with antibiotics/-mycotics to avoid bacterial or fungal contaminations.

#### 3.5.1 Maintenance in proliferation phase

Timing 30 min; incubation 7 days.

1. Change medium every 48–72 h, i.e., Monday, Wednesday, Friday.
  - Inspect macroscopically and microscopically for contamination.
  - Remove old medium and add 0.5 mL of fresh ProM into the insert on the apical part of the membrane and 4 mL in the well to the basal part.

#### 3.5.2 Maintenance in differentiation phase

Timing 30 min; incubation 28 days.

1. After 7 days of culture, remove ProM, and add 4 mL of DiffM (see 2.4.14) only to the basal side in the well, while leaving the apical side empty.
2. Change medium every 48–72 h, i.e., Monday, Wednesday, Friday:
  - Inspect macroscopically and microscopically for contamination.
  - Remove medium and add 4 mL of DiffM in the well to the basal side

Critical: It is essential that the epithelial cells reach confluency before entering the ALI phase. Due to the optical properties of the fibrin gel, visualizing the surface of the tri-culture requires an upright bright field microscope. If that is not available, perform pre-experiments under submerged conditions to estimate the culture time needed for a continuous epithelial layer.

Tip: We do not recommend using TEER (transepithelial electrical resistance) measurements to assess monolayer integrity. Hydrogel shrinkage and detachment from the insert wall might introduce small gaps between insert and hydrogel. As a result, the current leaks through the gaps and the resistance drops significantly.

Critical: Once the gels are in ALI, the lack of medium with antibiotics on the apical side will make the model more sensitive to contaminations. Take extreme precautions when changing medium, opening the dish as little as possible, moving the arms slowly and avoiding crossing or passing over them. Even when working in a laminar flow hood, avoid talking or coughing while handling the culture.

### 3.6 Termination of tri-culture and downstream analysis

The termination procedure is highly dependent on the desired downstream read-outs. It is possible to perform multiple evaluation methods per insert. Note that Section 3.6.1 requires an intact cell culture insert. Afterwards, the sample can be portioned strategically.

#### 3.6.1 Characterization of mucociliary clearance

The transport of mucus-entrapped fluorescent beads driven by ciliary beating on the luminal side of the mature tri-culture was recorded using an upright microscope equipped with a fluorescence unit. After addition of the beads, videos were recorded at room temperature. Particle trajectories and corresponding track mean speed was determined using the Fiji Plugin Trackmate [see Ershov et al., (2022)].

Timing 15 min.

1. Transfer cell culture insert from deep to standard 12-well plate with 500 µL of DiffM at the basal side of insert.
2. Add 50 µL of fluorescent bead suspension (see 2.4.15) to the apical side of cell culture insert.
3. Distribute beads evenly by gentle rocking of well plate.
4. Carefully remove excess bead suspension with a micropipette (a sufficient number of beads will remain on the sample surface).
5. Place well plate underneath upright microscope, remove lid.
6. Record videos/time series of moving fluorescent beads at an appropriate magnification.

Tip: This procedure is easiest to perform using an intact cell culture insert. Afterwards, the insert can be portioned strategically to proceed with other downstream applications.

Tip: Recordings should be carried out with a magnification that allows the distinction of single fluorescent particles.

Critical: Once the lid of the well plate is removed, sterility is no longer guaranteed. Consider fixing the tissue afterwards immediately or quickly proceed with ciliary beating frequency analysis.

Critical: Particles are often transported along global or local mucus swirls that circle around a center. Hence, particle position along the insert radius affects particle speed. To reproducibly compare field of interests from different samples, it might be crucial to only record videos/time series in the same radial range of all inserts.

#### 3.6.2 Removal of hydrogel from cell culture insert

Timing 15 min.

1. Take insert out of well plate using tweezers.
2. Remove membrane from insert using a pointy scalpel by slowly cutting along the edge of the membrane.
3. Place membrane plus attached hydrogel in glass Petri dish.
4. Divide hydrogel with round scalpel into different pieces, their size depends on the downstream application.

Critical: Fibrin gel and membrane separate easily from each other. This should be avoided to maintain structural support of the gel by the membrane. Once soft fibrin gels collapse, it is difficult to recover their original shape.

#### 3.6.3 Ciliary beating frequency analysis

Observation of real-time ciliary beating was performed using an inverted brightfield microscope equipped with a high-speed camera and a temperature incubation unit. Several fields of interest were recorded with a 60× objective at 32 °C. The beating frequency was analyzed using the Fiji plugin FreQ (https://github.com/hansenjn/FreQ/). This fast Fourier transform-based approach determines pixel-intensity oscillations in 2D images and summarizes the results in a heat map and a histogram of the ciliary beating frequency. [For detailed protocol see Jeong et al., (2022)].

Timing 30 min.

1. Transfer a small amount of DiffM into a chambered coverslip.
2. Place sample (at least one-quarter of sample) upside down into the medium.
3. Make sure that a thin film of medium is separating sample and glass.
4. Make sure that the sample does not move.
5. Transfer to microscope, preferably with a temperature-controlled incubation unit at 32 °C.
6. Find beating cilia in proximity to the bottom layer of the chambered coverslip.
7. Record up to 1,000 frames per location at a framerate of minimum 100 fps using a high-speed camera.

Critical: Although ciliary beating is stable for several hours after disassembly of the tissue, the beating frequency is affected by factors like temperature, pH and medium composition. To achieve reproducible results, these parameters need to be kept constant.

Tip: To make high-speed recordings of motile cilia with an inverted bright field microscope, it is beneficial to use a high magnification (40x or 60x objective). Since the light has to pass through the hydrogel, it might be necessary to increase the light source intensity. For best results, choose a frame rate of at least 100 fps. In general, a tradeoff between frame rate and resolution should be considered.

#### 3.6.4 Histology

After fixation using Carnoy’s fixative and dehydration (Table 2), overall tissue morphology and mucus production were assessed using the periodic acid-Schiff reaction with hematoxylin counter-staining. Moreover, cell-type-specific markers were localized with immunohistochemistry. The following markers were utilized: secretoglobin family 1A member 1 (SCGB1A1) typical for club cells, mucin 5AC (MUC5AC) found in goblet cells, transcription factor tumor protein p63 (TP63) typical for basal cells, keratin 5 (KRT5), a characteristic intermediate filament for basal cells, acetylated tubulin as marker for cilia and claudin 1 as tight-junction component to assess the epithelial barrier (Table 3).

**TABLE 2:** Dehydration series. *Paraffin should be heated to 60 °C*.

Step number	Solution	Concentration (diluted in ultrapure water)	Duration
1	Isopropanol	30%	1 h
2	Isopropanol	50%	1 h
3	Isopropanol	80%	1 h
4	Isopropanol	90%	1 h
5	Isopropanol	100%	1 h
6	Isopropanol	100%	1 h
7	Xylene	100%	1 h
8	Xylene	100%	1 h
9	Paraffin*	100%	1 h
10	Paraffin*	100%	3 h

**TABLE 3:** List of antibodies for immunofluorescence.

Marker	Description	Species	Company, Cat. num	Dilution
ACE2	Angiotensin Converter Enzyme 2, expressed in both epithelial and endothelial cells. Receptor of the respiratory SARS-CoV-2 virus (Zhang et al., 2020)	Goat	RnD Systems, AF933-SP	1:20
Acetylated tubulin	Component of centrioles and cilia (Schamberger et al., 2015)	Mouse	Sigma-Aldrich, T7451	1:800
SCGB1A1	Secretoglobin family 1A member 1, also known as Uteroglobin, is a marker for the progenitor, mucus-producing cell type known as club cell (Okuda et al., 2019)	Rabbit	Invitrogen, PA5-95864	1:50
PECAM1	Also known as platelet adhesion molecule (CD31), PECAM1 serves as a marker for endothelial cells (Müller et al., 2002)	Mouse	Sigma-Aldrich, P8590	1:200
Claudin 1	Tight junction, maintaining the epithelial barrier (Kaarteenaho et al., 2010; Schlingmann et al., 2015)	Rabbit	Biorbyt, orb214856	1:800
FOXJ1	Forkhead box protein J1, transcription factor and regulator of ciliated cell differentiation (Zou et al., 2023)	Rabbit	Invitrogen, PA5-52189	1:500
KRT5	Keratin specific for basal stem cells (Rock et al., 2009)	Rabbit	Abcam, ab52635	1:200
MUC5AC	Glycoprotein mucin 5AC found in goblet cells and on the luminal side of the airways, but absent in the submucosal glands (Okuda et al., 2019)	Mouse	OriGeneAM 50143PU-T	1:800
MUC5B	Glycoprotein mucin 5B present in both the epithelial secretory cells and the submucosal glands (Okuda et al., 2019)	Rabbit	Invitrogen, PA5-82342	1:500
Pan-cytokeratin	Broad marker of all cytokeratin proteins, intermediate filaments present in all respiratory epithelial cells (Maestre-Batlle et al., 2017)	Rabbit	Acris, AM33270RP-N	1:200
PAK1/PAK2/PAK3	P21-activated kinases 1/2/3 regulate, among others, cilia formation (Gonzalez-Rubio et al., 2023)	Rabbit	Cell Signalling Technology, 2601T	1:100
TP63	Member of the p53 family and marker of stemness in respiratory basal cells (Warner et al., 2013)	Mouse	Santa Cruz, sc-25268	1:50

##### 3.6.4.1 Fixation using Carnoy’s fixative

Timing 1.5 h.

1. Place sample (at least one-quarter) in embedding cassette, protected between two sponges.
2. Transfer cassette to glass beaker with Carnoy’s fixative (see 2.4.16).

Caution: Carnoy’s solution is toxic when inhaled. Work under a chemical hood with proper ventilation and adequate personal protective equipment.

1. Incubate for 1 h at room temperature.
2. Place cassette in 100% ethanol for 15 min.
3. Repeat previous step with fresh ethanol.

##### 3.6.4.2 Agarose embedding

Timing 1 h.

1. Add warm 2% agarose solution (see 2.4.17) to cryomolds.
2. Quickly transfer specimens from cassette into plastic cryomolds, cover completely with agarose.
3. Let agarose harden for 30 min at 4 °C.
4. Remove agarose from cryomolds using a scalpel.
5. Place agarose-embedded specimen back in tissue cassette.
6. Samples can now be stored in 70% ethanol at 4 °C for up to 1 month.

##### 3.6.4.3 Dehydration and paraffin embedding

Timing 24 h.

1. Proceed with dehydration series by washing the samples in the cassettes according to Table 2.
2. After dehydration, samples can remain in warm paraffin (60 °C) for a maximum of 12 h.
3. Move cassettes to a paraffin embedding station.
4. Embed each sample in a paraffin block following the station’s manufacturer guidelines.
5. Cut and stain 3 µm paraffin sections using standard histology protocols for periodic acid-Schiff reaction and/or immunohistochemistry. A list of validated antibodies can be found in Table 3.

#### 3.6.5 Whole-mount immunostaining

To visualize three-dimensional vascular structures inside the samples, whole-mount immunostaining of the endothelial cell marker PECAM1 (platelet and endothelial cell adhesion molecule 1) and the mesenchymal filament vimentin (Table 4) was performed. Stained specimens were imaged with a fluorescent microscope with a high Z-axis depth and resolution such as confocal or multiphoton. Vascular structures were analyzed in either 2D using a maximum intensity projection (MIP) of the Z-axis, or in 3D with a segmentation software such as IMARIS 10 (Oxford Instruments).

**TABLE 4:** List of tested antibodies for confocal/multiphoton microscopy.

Marker	Description	Species	Company, Cat. num	Dilution
PECAM1	Also known as platelet adhesion molecule (CD31), PECAM1serves as a marker for endothelial cells (Müller et al., 2002)	Mouse	Sigma-Aldrich, P8590	1:200
Vimentin	Intermediate filament expressed in mesenchymal cells	Mouse	Sigma-Aldrich, 180052	1:100

##### 3.6.5.1 Fixation using methanol

Timing 15 min.

1. Transfer sample (at least one-quarter) to new 48-well plate wells.
2. Fix sample by adding 250 µL of ice-cold methanol to each well.
3. Incubate for 10 min at −20 °C.

Caution: Methanol is toxic and a teratogen. Work under a chemical hood with proper ventilation and adequate personal protective equipment.

##### 3.6.5.2 Immunostaining

Timing 5 days.

1. Dilute primary antibody (see Table 4) in blocking solution (see 2.4.18).
2. Add 250 µL of the primary antibody solution to the samples.
3. Fill remaining wells with PBS and wrap the plate using Parafilm to reduce evaporation.
4. Incubate at 37 °C for 48 h. A cell culture incubator can be used, but the CO_2_ concentration is irrelevant.

Critical: Antibodies are large molecules with a slow diffusion rate through dense hydrogels. It is therefore essential to have long incubation times to allow the homogenous staining of the whole sample.

1. Remove primary antibody solution from the wells.
2. Wash twice with 500 µL PBS for 5 min each.
3. Wash a third time with 500 µL PBS for at least 8 h (e.g., overnight) at 37 °C.
4. Choose a secondary antibody against the primary antibody species, coupled to a fluorophore of desired excitation/emission wavelength based on the microscope specifications (e.g., Goat anti-Mouse Alexa Fluor 594, Invitrogen).
5. Dilute the secondary antibody in blocking solution based on the manufacturer’s information (e.g., 1:400).
6. Add 250 µL of the secondary antibody solution in each of the wells.
7. Incubate at 37 °C for 48 h.
8. Remove secondary antibody solution.
9. Wash each well three times with 500 µL PBS for 5 min each.
10. Prepare a DAPI solution diluting 7.5 µL DAPI stock solution in 5 mL PBS
11. Add 250 µL of DAPI solution in each well.
12. Incubate 15 min at room temperature.
13. Remove DAPI solution.
14. Wash 3 times for 5 min each with PBS.
15. Close plate with Parafilm.
16. Wrap well plates in aluminum foil to protect the fluorophores from light.
17. The plates can be stored at 4 °C for several weeks, but fluorophores will degrade over time. It is recommended to image the samples within 5 days after the staining.
18. For long-term storage, use PBS supplemented with 1% ABM to avoid contamination of the samples.

#### 3.6.6 Scanning/transmission electron microscopy

The presence of cilia was confirmed using scanning and transmission electron microscopy. Samples were fixed with 3% glutaraldehyde and afterwards treated according to standard protocols for scanning/transmission electron microscopy [briefly described here Luengen et al., (2022)].

Timing 15 min.

1. Place sample (at least one-quarter) in 3% glutaraldehyde diluted in Sorensen’s phosphate buffer. Store at 4 °C for up to 2 months.
2. Process samples according to standard protocols for scanning/transmission electron microscopy.

#### 3.6.7 RNA isolation

Total RNA was isolated to perform gene expression analysis. RNA from both epithelial cells and gel-entrapped endothelial and stromal cells was harvested using a guanidinium thiocyanate-phenol-chloroform extraction. Prior to that, the samples were mechanically dissociated to ensure complete cell lysis.

##### 3.6.7.1 Mechanical dissociation

Timing 30 min.

1. Transfer sample (or at least one-third) plus membrane to sterile 1.5 mL tube with 250 µL of TRIzol/TRI Reagent.
2. Thoroughly dissociate hydrogel with nuclease-free 1 mL tip or sterile scalpel.
3. Add 750 µL of TRIzol/TRI Reagent to suspension.
4. Incubate for 15 min at room temperature to allow complete cell lysis.
5. Samples treated with TRIzol/TRI Reagent can be stored at −80 °C up to several months.

Caution: Exposure to ingredients of TRIzol/TRI Reagent like phenol and guanidinium thiocyanate can lead to serious health problems. Read the safety datasheets and use a chemical hood and personal protection equipment.

##### 3.6.7.2 RNA isolation

Timing 2 h.

1. Add 0.2 mL of chloroform per 1 mL of TRIzol/TRI Reagent.
2. Mix both phases by vortexing for 15 s.
3. Incubate mixture for 15 min at room temperature.
4. Centrifuge for 15 min at 12,000 g at 4 °C, remaining fibrin residues will collect in the lower phase at the bottom of the tube.
5. Transfer aqueous phase to new RNAse-/DNAse-free 1.5 mL tube.
6. Add 0.5 mL of isopropanol per 1 mL of TRIzol/TRI Reagent.
7. Incubate for 10 min at room temperature.
8. Centrifuge precipitated RNA for 10 min at 12,000 g at 4 °C.
9. Remove supernatant carefully.
10. Add 1 mL of 70% EtOH to RNA pellet.
11. Centrifuge at 7,500 *g* for 5 min at 4 °C
12. Remove supernatant carefully.
13. Repeat step 127. - 129. twice.
14. Air-dry RNA pellets at 55 °C.
15. Depending on the size, dissolve the pellet in an appropriate amount of nuclease-free water (e.g., 15–60 µL).
16. Determine RNA concentration and purity with spectrophotometry.
17. For long-term storage, keep isolated RNA at −80 °C.

Tip: Repeating the washing step with 70% EtOH will improve RNA purity.

Critical: Carefully monitor the air-drying process of the RNA pellet to prevent over-drying. Otherwise, water solubility of the RNA will be reduced significantly.

### 3.7 Timing overview

Steps 1–13: Primary human endothelial cell culture.
  Steps 1–5: Coating of cell culture flasks with gelatine, 45 min.
  Steps 6–11: Thawing of endothelial cells, 20 min.
  Steps 12–13: Expansion of endothelial cells, 1 week.
Steps 14–21: Primary human stromal cell culture.
  Steps 14–19: Thawing of stromal cells, 20 min.
  Steps 20–21: Expansion of stromal cells, 1 week.
Steps 22–49: Preparation of cell-laden hydrogel.
  Steps 22–24: Preparation of hydrogel components, 30 min.
  Steps 25–34: Preparation of cell suspensions, 1.5 h.
  Steps 35–41: Hydrogel preparation, 1.5 h.
  Steps 42–49: Seeding of respiratory epithelial cells, 30 min.
Steps 50–52: Maturation of tri-culture.
  Step 50: Maintenance in proliferation phase, 1 week.
  Steps 51–52: Maintenance in differentiation phase, 4 weeks.
Steps 53–134: Termination of tri-culture and downstream analysis.
  Steps 53–58: Characterization of mucociliary clearance, 15 min.
  Steps 59–62: Removal of hydrogel from cell culture insert, 15 min.
  Steps 63–69: Ciliary beating frequency analysis, 30 min.
  Steps 70–85: Histology, 24 h.
  Steps 86–110: Whole-mount immunostaining, 5 days.
  Steps 111–112: Scanning/transmission electron microscopy, 15 min.
  Steps 113–134: RNA isolation, 1.5 h.

## 4 Anticipated results

### 4.1 The tri-culture replicates histology and functionality of the airway mucosa

**FIGURE 2:**
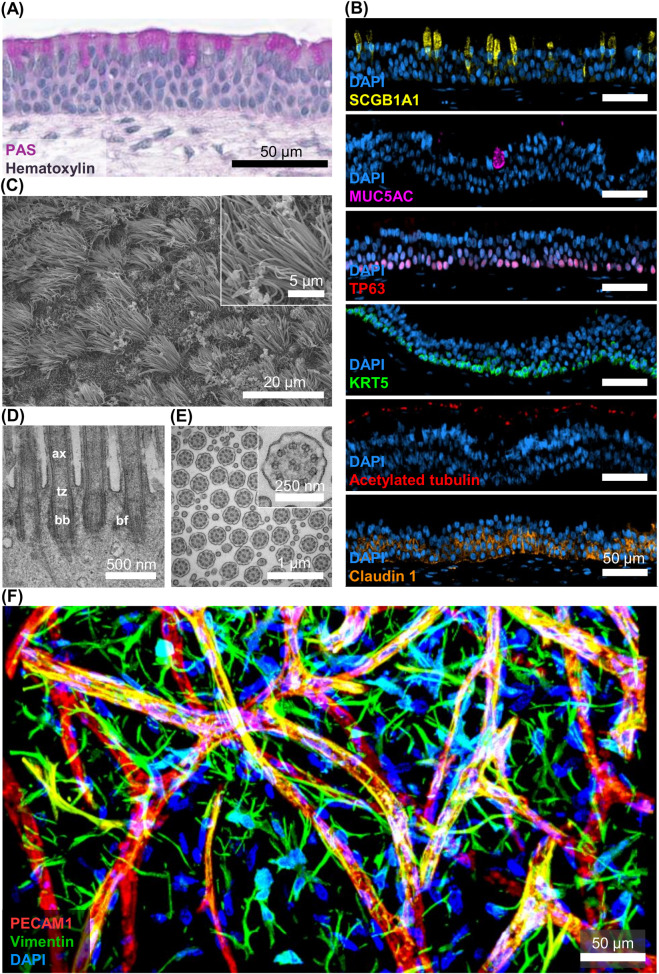
Mucociliary differentiation of tracheal airway epithelial cells and formation of vascular structures after 4 weeks of culture at air-liquid-interface. **(A)** Representative microscopic image of tri-culture stained with Periodic Acid-Schiff Reaction (PAS) - hematoxylin. **(B)** Representative immunofluorescent images of cell-type-specific markers, from top to bottom: Club cells (SCGB1A1), goblet cells (MUC5AC), basal cells (TP63 and KRT5), ciliated cells (Acetylated Tubulin), tight junctions (claudin 1) [adapted from (Gonzalez-Rubio et al., 2023)]. **(C)** Scanning electron microscopy images of ciliated surface of tri-culture. **(D)** Longitudinal section of multiciliated epithelial cell visualized by transmission electron microscopy. Structural parts of cilia indicated by abbreviations: ax = axoneme, bb = basal body, tz = transition zone, bf = basal foot. **(E)** Transversal section of multiple cilia demonstrating the central microtubule pair surrounded by 9 doublets (9 + 2 configuration). **(F)** Projection of a 3D stack made by two-photon laser scanning microscopy of a tri-culture fibrin hydrogel with endothelial vessels (PECAM1, red) and stromal cells (vimentin, green) [adapted from (Luengen et al., 2022)].

After 35 days of culture, the combination of initially naive epithelial, endothelial and stromal cell types successfully recapitulates the histology of the human airway mucosa (Figure 2). To assess general features and mucus production, a histological staining combining the periodic acid-Schiff reaction and hematoxylin was performed. Figure 2A shows the typical high-columnar pseudostratified cell arrangement, characterized by a single layer of cells with nuclei located at different heights. A more detailed evaluation of the cell composition was revealed by immunohistochemistry (Figure 2B). The respiratory epithelium is predominantly made of 3 cell types: secretory, ciliated and basal cells. In this model, secretory club cells are abundantly found within the epithelial layer. They are characterized by their high-columnar shape and localization of the marker SCGB1A1 throughout the basal-apical axis. On the other hand, mucus-containing goblet cells are among the rarer cell types of the airway epithelium and were characterized by the expression of the mucin MUC5AC. They are round in shape and filled with mucus granules. In addition, MUC5AC expression is often observed in the mucus layer overlying the epithelium. Basal cells were detected by expression of their respective markers, TP63 and KRT5. Figure 2B shows the nuclear localization of the transcription factor TP63 in basal cells, as well as the cytosolic expression of intermediate filament KRT5. Ciliated cells make up a significant portion of the layer, as indicated by the staining of acetylated tubulin (Figure 2B). High-magnification images of cilia were recorded using scanning electron microscopy (SEM) and transmission electron microscopy (TEM). They reveal the ciliated surface area of the tri-culture (Figure 2C) and the detailed structural anatomy of single cilia (Figures 2D,E). The elongated axoneme (ax), which protrudes from the cell membrane, is visible in the longitudinal TEM section. The axoneme is connected to the basal body (bb) via the transition zone (tz). On the lateral side of the basal body, the cone-shaped basal foot (bf) is localized (Figure 2D). Transversal sections demonstrate the typical microtubuli arrangement of motile cilia, which comprises a central pair of microtubuli surrounded by nine doublets (Figure 2E). Moreover, transverse TEM images provide information on the axonemal and basal body orientation. These are useful indicators to determine the ciliary beating direction as well as rotational and tissue-level polarity (Lyu et al., 2023). Figure 2F shows the vascular structures formed by endothelial cells after 28 days of culture. This interconnected network is supported by the surrounding stromal cells, which secrete pro-angiogenic factors and guide the vascularization.

**FIGURE 3:**
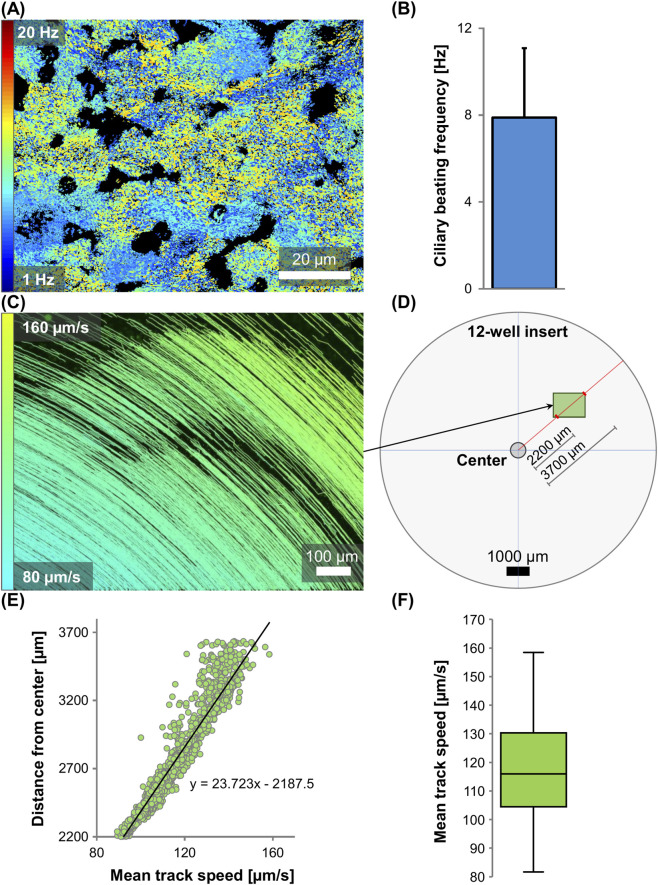
Ciliary beating frequency and particle transport of tri-culture differentiated for 4 weeks at air-liquid interface. **(A)** Heatmap of ciliary beating frequency composed of colored signal pixels with frequency values between 3 and 15 Hz. Corresponding brightfield live cell imaging was recorded with 224 fps at 32 °C, see Supplementary Video 2. **(B)** Representative mean ciliary beating frequency ± SD: 7.9 ± 3.2 Hz. **(C)** Particle trajectories of fluorescent beads transported by ciliary beating. Live-cell imaging recorded at room temperature, see Supplementary Video 3. Mean track speed between 80 and 160 μm/s is represented by respective track coloring. **(D)** Representative map of 12-well insert indicating the distance between the field of view from **(C)** and the center of the insert along the insert radius. **(E)** Distance of tracks from center of cell culture insert plotted over mean track speed with fitted regression line and formula. **(F)** Boxplot presenting global mean track speed of particle trajectories, median 115.9 μm/s.

Besides ciliary anatomy, ciliary functionality is a second important parameter that can be assessed using the tri-culture. The ciliary beating frequency (CBF) was determined using high-speed recordings of real-time ciliary beating (see Supplementary Video 2). Figures 3A,B show a representative heatmap with colored signal pixels and the corresponding average frequency (mean ± SD: 7.9 ± 3.2 Hz). Generally, the average CBF of the tri-culture epithelium is expected to be within the range of 6–8 Hz. This is in accordance with results reported in healthy *ex vivo* multiciliated cells and other airway *in vitro* models (Sommer et al., 2010; Zhou et al., 2019; Tratnjek et al., 2020). Moreover, the tri-culture exhibits a functional mucociliary clearance activity. This process requires the formation of a mucus layer that is mobilized by coordinated ciliary beating. To investigate this phenomenon, the movement of mucus-entrapped fluorescent beads was recorded and tracked (see Supplementary Video 3). Figure 3C demonstrates representative particle trajectories. Mean track speed between 80 and 160 μm/s is indicated by respective track coloring. The corresponding global track speed (median 115.9 μm/s) is shown in Figure 3F. However, due to circular mucus swirls, particle velocity is often dependent on the radial location within the insert (see Figure 3D). A more appropriate method of defining clearance activity is to report the relationship of particle distance from the insert center and speed (Figure 3E). Although the direct comparison to healthy human airways is difficult, a general clearance rate of 4–20 mm/min (=66.7–333.3 μm/s) is expected (Sakakura et al., 1983; De España et al., 1986; Yeates et al., 1975; Cruz et al., 1974).

### 4.2 Limitations and troubleshooting

We successfully produced tri-cultures with human airway epithelial cells of nasal, tracheal and bronchial origin. Although primary cells demonstrate superior differentiation capacities in comparison to respiratory cell lines (Bukowy-Bieryłło, 2021; Lodes et al., 2020), they also have limitations. Availability of healthy primary human airway tissue is limited, and differentiation into a mucociliary phenotype is highly dependent on multiple factors. Cells from nasal, tracheal or bronchial portions of the airway require customized isolation protocols and media compositions. For example, we demonstrated that differences in growth factor concentrations directly influence cell behavior (Luengen et al., 2020). In addition, the quality of differentiation is affected by the passage number of airway epithelial cells. Declining differentiation potential with increasing passage is a well-described phenomenon. In our experience, the best results are achieved with cells in passage 1. However, this factor is highly individual, as reported by other groups (Rayner et al., 2019; Leung et al., 2020; Myo et al., 2025). Another common drawback of primary cells is donor-to-donor variation. So far, there is no established workflow to identify non-differentiating epithelial cell lines other than the time-consuming maturation at air-liquid interface. We recommend regular screening of donors using 2D or 3D ALI protocols, followed by assessment of basal, ciliated and secretory cells. Interestingly, epithelial cells differentiate more robustly in the presence of stromal supporting cells, reducing the degree of donor failure (Albers et al., 2016; Luengen et al., 2022). We obtained best results using bone marrow- and trachea-derived stromal supporting cells. However, stromal cells from nasal resections or fat tissue were less potent in promoting epithelial differentiation or formation of vascular-like structures (Luengen et al., 2022; Gonzalez-Rubio et al., 2023). Also, not only origin, but cell identity of the stromal cell population highly influences vascularization (Gonzalez-Rubio et al., 2025). We achieved robust *in vitro* vascularization using human umbilical vein endothelial cells (HUVECs), especially when employed in early passages and in combination with the appropriate type of supporting cells (Kniebs et al., 2020).

The here described procedure is not limited by the requirement for highly specialized training, equipment or facility. However, advanced skills in handling and maintaining highly susceptible cell types are advantageous. In addition, long culture periods increase the risk for contamination massively. This protocol does not accelerate the mucociliary differentiation process and employs the gold standard of maintaining cultures at ALI for 4 weeks. Therefore, additional resources, such as separate incubators, can be beneficial. Nevertheless, pitfalls can be avoided. For a detailed troubleshooting guide, see Table 5.

**TABLE 5:** Troubleshooting.

Step	Problem	Possible reason	Solution
12–13	Endothelial cells are not growing and/or showing abnormal morphology	Endothelial cells express senescence by losing their cobblestone shape, taking on a “fried egg” morphology, and by slowing down their division. If this is observed, cells need to be discarded since it will usually imply a lack of vascularization potential within the hydrogel Factors include (1) use of high passages; (2) long culture time in almost full confluency; (3) lack of gelatine coating in the flasks; or (4) donor-specific problems	For optimal endothelial cell growth conditions: (1) prevent cells from becoming too confluent by closely observing their growth and perform passages at 70% confluency; (2) do not use cells in high passages; (3) eliminate donors which fail to proliferate or only at a very low speed
26–28	Cells are not detaching	(1) Decreased potency of trypsin because of repeated warming-up of the reagents; or (2) cells were cultured until they reached over 70% confluency	(1) Use a new trypsin batch; (2) confirm that PBS is free of calcium and magnesium; (3) detach cells before they become confluent; (4) opt for up to 15 min of incubation time in PBS before adding trypsin. (5) opt for longer trypsin incubation
6–11, 14–19, 42–45	High cell death after thawing	(1) Cells were stored too long at −80 °C after freezing and before the storage in liquid nitrogen; (2) the cells were thawed briefly during storage or transportation; or (3) thawing was not fast enough before seeding	(1) Provide proper long-term storage conditions, best at temperatures between −135 °C and −190 °C; (2) ensure that cell transports proceed without accidental thawing of vials; (3) follow cell thawing protocol closely and do not increase the incubation time at 37 °C inside water bath
39	Gel does not polymerize	(1) Fibrinogen polymerization reaction is not optimized with regard to free calcium ion concentration or pH value; (2) fibrinogen or thrombin were not stored at optimal temperature or experienced freeze-thawing cycles	(1) Make sure no phosphates (e.g., PBS) are added to the mixture; (2) check pH (if TBS is prepared from 10x stock solution consider that ultrapure water is acidic, and pH will need to be adjusted); (3) do not store fibrinogen or thrombin at room temperature or 4 °C since their stability is low when not frozen; (4) use new batches of the components
35–38	Difficulties casting the gels	(1) Preparation of fibrin gels was performed at room temperature, which speeds up the polymerization process. If the solutions are not mixed properly, gels polymerize inhomogeneously	(1) Cool down solutions to slow fibrin polymerization by placing all solutions on ice before usage
51–52	Gel is degrading too fast	(1) No addition of tranexamic acid or insufficient final concentration in growth medium; (2) when using an immortalized bronchial cell line, we already observed fibrin degradation despite addition of 0.16% TXA.	(1) Check tranexamic acid concentration and consider increasing the concentration; (2) change the utilized cell type/donor, since some cell lines degrade fibrin faster than others
51–52	Accumulation of mucus on apical surface of fibrin gel	(1) During the 4 weeks of ALI culture, mucus accumulates inside the cell culture insert. The extent of mucus production can vary between epithelial cells of different donors	(1) Wash apical surface of fibrin gels by carefully adding 0.5 mL of DiffM to the insert and incubate for 15 min at 37 °C. Afterwards, carefully remove again. This procedure can be repeated up to two times per week during the differentiation phase
51–52	Epithelial cells are not generating cilia	(1) High passage numbers of respiratory epithelial cells; (2) improper handling of sensitive cell types during thawing or culture; (3) starting the differentiation phase in ALI before epithelial cells are fully confluent; (4) usage of expired media or media with degraded ingredients	(1) Use a lower passage; (2) pay special attention to the handling of sensitive cell types like primary respiratory epithelial cells (decrease speed of pipet controller/centrifuge); (3) make sure epithelial cells are confluent before switching to ALI; (4) only warm up medium in ready-to-use aliquots to reduce growth factors degradation
51–52	Lack of vascularization in the gel	(1) If endothelial cells are not forming vessels, they might have entered senescence; (2) inadequate mechanical properties of fibrin hydrogel	(1) Use endothelial cells with a lower passage; (2) make sure they never reached full confluency when cultured before use; (3) try different donors; (4) use a different supporting cell type; (5) only warm up medium in ready-to-use aliquots to reduce growth factors degradation; (6) Fine-tune mechanical properties of hydrogel by varying final fibrinogen concentration
85, 89–110	Immunofluorescence not working	(1) Antibody is not specific for antigen; (2) insufficient incubation time of antibody solution	(1) Check the antibody specificity with a positive control; (2) increase incubation time of antibody solution to allow a better diffusion of the antibody solution

## 5 Discussion

While the vascularized 3D tri-culture model is a more recent development, the culture of respiratory epithelial cells at air-liquid interface is a widely applied method, which has been used for several decades (Choe et al., 2006; Gruenert et al., 1995). However, the herein described model demonstrates superior potential for application in airway research. This mainly depends on two factors: the exclusive use of primary cells and the organotypic combination of multiple cell types in a 3D matrix. Both are determining factors for the close anatomical and physiological resemblance to the *in vivo* counterpart.

Traditionally, many airway *in vitro* models employ tumor or immortalized cell lines (Baldassi et al., 2021; Silva et al., 2023). Although this enables larger sample sizes, heterogeneity, functionality and responsiveness are compromised. As proven with our tri-culture, a well-matured layer of respiratory epithelial cells exhibits a pseudostratified morphology with the expression of differentiation markers for basal, goblet, club and ciliated cells (Figures 2A–C). This is accompanied by luminal mucus production and generation of functional cilia. Some cell lines like the bronchial Calu-3 cells or the human bronchial epithelial cell line HBEC3-KT show expression of the mucin proteins MUC5AC and MUC5B (Lodes et al., 2020; Meindl et al., 2015). In contrast to that, many other models like the bronchial cell line BEAS-2B or VA10 cells completely lack mucus production (Lodes et al., 2020; Stewart et al., 2012). Most remarkably, not one immortalized cell line has been reported to reproducibly generate functional ciliation in a recent study. The bronchial cell line HBECS3-KT has been shown to differentiate into a preliminary ciliary phenotype. However, with less than 5% of ciliated cells covering the model’s surface, no functional ciliary clearance is provided (Lodes et al., 2020). In primary respiratory epithelial cells, many studies report the ciliation of differentiated epithelial cells *in vitro* (Rayner et al., 2019; Leung et al., 2020; Prytherch et al., 2011). However, despite mucus expression and cilia formation, many monolayers generated from primary cells still demonstrate resemblance with simple cuboidal or stratified squamous epithelial arrangements (Leung et al., 2020; Prytherch et al., 2011). This is significantly improved by the presence of a cell-laden hydrogel underneath the epithelium. The embedding of supplementary cell types enables crucial cellular crosstalk, which is relevant for many pathological conditions such as the development of idiopathic pulmonary fibrosis or the tissue repair response after cigarette smoke exposure (Barron et al., 2024; Iskandar et al., 2015). We demonstrated enhanced differentiation of tracheal airway epithelial cells in co- and tri-culture with supporting stromal and endothelial cells compared to monocultures (Luengen et al., 2022; Albers et al., 2016). In addition, the rate of donor failures during differentiation of epithelial cells from nasal origin was much lower in tri-cultures, compared to monocultures (Luengen et al., 2020; Kreimendahl et al., 2019). This observation is supported by others. For example, the cultivation of respiratory epithelial cells on top of a hydrogel scaffold enriched with lung fibroblasts suggested a co-culture-dependent increase in epithelial height and expression of differentiation markers (Leach et al., 2023). Furthermore, the implementation of endothelial cells is central to achieving relevant airway *in vitro* models. It is well established that the role of organotypic vasculature not only involves passive transport of nutrients and circulating cells. Instead, endothelial cells dynamically shape the tissue microenvironment and engage in cellular crosstalk (Augustin and Koh, 2017). Still, many complex airway *in vitro* models do not include endothelial cells. Other approaches utilize two-dimensional monolayers of endothelial cells cultured on the basal side of cell culture inserts to investigate endothelial-epithelial crosstalk (Viola et al., 2021; Iriondo et al., 2025). While this experimental setup gave insights into the effect of intercellular signaling on barrier formation and processing of inflammatory stimuli (Blume et al., 2017), it fails to recapitulate the three-dimensional microvasculature found in the native airway mucosa. The respective assembly of *in vitro* vascular networks requires a 3D matrix loaded with both endothelial and supporting cells. However, the establishment of *in vitro* vascularization comprises several aspects. Formation of vascular structures is affected by the choice of stromal supporting cells. We found that both stromal cell origin and identity affect metrics like vessel volume and length (Kniebs et al., 2020; Gonzalez-Rubio et al., 2025). Moreover, our tri-culture model showed that the additional presence of airway epithelial cells also significantly increases vessel branching points in comparison to the respective co-cultures (Luengen et al., 2022). The characterization of vascular structures is performed using 3D imaging (e.g., confocal or multiphoton microscopy). The resulting volumetric dataset allows for the analysis of 3D vessel properties such as volume, length and sphericity. In addition, it provides information regarding the proximity and configuration of supporting cells with and around the formed vessels. Also, transmission electron microscopy is useful to reveal the structural details of the basement membrane, which acts as a stromal-endothelial interface (Gonzalez-Rubio et al., 2025). *In vitro* vascularization also relies on the mechanical and physical properties of the employed substrate, as well as the biological activity (Mastrullo et al., 2020). The 5 mg/mL fibrinogen hydrogels described here are reported to have a stiffness ranging from 0.4 to 3.4 kPa (Duong et al., 2009; Mooney et al., 2010). This range is the result of different methodologies used for assessing mechanical properties, as well as varying concentrations of thrombin and CaCl_2_, and polymerization times. Measuring the stiffness of native sub-epithelial tissue is challenging, and little information is available. However, two studies report stiffness of airway extracellular matrix at approximately 0.5 kPa and stiffness of healthy bronchial biopsies between approximately 3.5 and 15 kPa (Jamieson et al., 2021; Zemła et al., 2018). Thus, the stiffness of the here applied fibrin gel is in the range of that of healthy human airways. Also, unlike synthetic hydrogels, the viscoelastic and strain stiffening behavior of fibrin reflects crucial mechanical properties of living tissues (Janmey et al., 2009; Piechocka et al., 2010; Chaudhuri et al., 2016). Cell behavior is also affected by hydrogel degradability (Brusatin et al., 2018). The non-covalent nature of thrombin-initiated fibrinogen crosslinks promotes 3D cell spreading, in comparison to hydrogels with high density of covalent bonds, which require energy-consuming enzymatic cleavage (Soon et al., 2010; Caliari et al., 2016; Khetan et al., 2013). However, under homeostatic conditions, native airway extracellular matrix is predominantly composed of collagens and does not contain fibrin (Hoffman et al., 2023). Other 3D airway *in vitro* models have already applied scaffolds made from lung-derived ECM hydrogels or collagen (de Hilster et al., 2024; Ishikawa et al., 2017; Iriondo et al., 2025). In our experience, *in vitro* vascularization and epithelial differentiation is improved using fibrin in comparison to other natural hydrogels such as collagen or agarose (Kreimendahl et al., 2019; Kniebs et al., 2020). This is likely due to the roles of fibrin and fibrinogen during wound healing orchestration. By engaging in molecular and cellular interactions, they stimulate cytokine expression, proliferation and adhesion in multiple cell types, including endothelial cells and fibroblasts (Mosesson, 2005; Laurens et al., 2006). These intrinsic biological properties make fibrin an ideal candidate for 3D *in vitro* tissue engineering (Ahmed et al., 2008).

## 6 Conclusion

Here, we provide detailed step-by-step instructions for generating a three-dimensional, vascularized human airway model, which comprises three primary human cell types and a fibrin hydrogel scaffold. The mature tissue reconstitutes both phenotypic and functional characteristics of the native mucosa. These include a pseudostratified epithelium that produces secretory and multiciliated cells, as well as an interconnected network of vascular structures. The model offers a more appropriate platform for a multitude of applications in the field of airway research, compared to common one-dimensional and simplified models. We also demonstrate that relevant *in vitro* modelling can be achieved using standard laboratory procedures. In doing so, we want to encourage and support the advancement of reproducible and much-needed airway *in vitro* models.

## Data Availability

The raw data supporting the conclusions of this article will be made available by the authors, without undue reservation.
